# Advanced Secondary Intention Healing for Complex Soft-Tissue Defects Using Reprocessed Micronized Acellular Dermal Matrix

**DOI:** 10.3390/life14111479

**Published:** 2024-11-14

**Authors:** Ha Jong Nam, Dong Gyu Kim, Je Yeon Byeon, Da Woon Lee, Jun Hyuk Kim, Se Young Kim, Hwan Jun Choi

**Affiliations:** 1 Department of Plastic and Reconstructive Surgery, Soonchunhyang University Gumi Hospital, Gumi 39371, Republic of Korea; namssi3@naver.com (H.J.N.); soulloop@naver.com (S.Y.K.); 2 Department of Plastic and Reconstructive Surgery, Soonchunhyang University Bucheon Hospital, Bucheon 14584, Republic of Korea; 121883@schmc.ac.kr; 3 Department of Plastic and Reconstructive Surgery, Soonchunhyang University Cheonan Hospital, Cheonan 31151, Republic of Korea; 115954@schmc.ac.kr (J.Y.B.); 103022@schmc.ac.kr (D.W.L.); psdoctor@schmc.ac.kr (J.H.K.)

**Keywords:** complex wounds, secondary intention, micronized acellular dermal matrix, dressing material, wound healing

## Abstract

Secondary intention healing offers an alternative when surgical options are infeasible. This study analyzed the effect of micronized acellular dermal matrices (mADMs; CGderm Matrix^®^, CG Bio, Seoul, Republic of Korea) on secondary intention healing in patients with complex soft-tissue defects and assessed mADMs’ efficacy in promoting secondary healing and improving clinical outcomes in these challenging cases. This retrospective study included 26 patients treated with sheet-type reprocessed mADMs between August 2022 and December 2022 at Soonchunhyang University Cheonan Hospital. Patients with full-thickness skin defects classified as complex wounds were included. Data on demographics, wound characteristics, and treatment outcomes were collected and analyzed. Wound area was measured using ImageJ software, and statistical analyses were conducted using SPSS. The application of mADMs resulted in a median wound area reduction of 81.35%, demonstrating its significant efficacy in wound healing. Most patients presented with compromised vascular supply, significant tissue loss, or infections that precluded conventional surgical interventions. No significant correlations were observed between patient variables and wound-healing outcomes, indicating the complex nature of wound healing. mADMs effectively promote secondary intention healing by providing a supportive extracellular matrix scaffold that enhances epithelialization and angiogenesis. Their rapid absorption, ease of handling, and ability to improve wound tensile strength make them particularly suitable for complex wounds.

## 1. Introduction

Complex skin and soft-tissue defects present significant challenges in wound management. The reconstruction of these wounds often involves various surgical methods; however, these options are not always feasible due to the patient’s condition or other constraints. In such cases, secondary intention healing may serve as an alternative. Secondary intention is a wound healing process which occurs when a wound is left open to heal naturally without surgical closure. The wound is gradually filled with new tissue, contracts over time, and eventually closes. This process can be influenced by several factors, including wound size, depth, location, presence of infection, patient age, general health, nutritional status, smoking status, and perfusion to the affected area [1]. The state of perfusion is crucial for successful healing especially in the lower extremities [2].

Recent advancements in wound-healing therapies have focused on promoting early healing at the molecular level. Among these, acellular dermal matrices (ADMs) have shown significant potential for accelerating the healing process. ADMs stimulate angiogenesis and provide a scaffold for granulation tissue formation, facilitating wound healing by promoting cell migration and revascularization [3,4,5]. ADMs are typically used as dermal substitutes for skin grafts. However, skin grafts may not be necessary or feasible in many cases because of the patient’s condition or other circumstances. In such scenarios, ADMs can be directly applied to the wound to promote epithelialization or reduce wound size through secondary intention healing [6].

Various ADM products have been developed and widely used in clinical settings to promote tissue regeneration and wound healing. Examples include Kerecis^®^ (piscine skin-derived, Kerecis Corp., Isafjordur, Iceland), AlloDerm^®^ (human skin-derived, LifeCell Corp., Branchburg, NJ, USA), Integra^®^ (bovine tendon type I collagen, Integra Lifesciences, Princeton, NJ, USA), and EZ Derm^®^ (porcine aldehyde cross-linked dermal collagen, Molnlycke HealthCare, Gothenburg, Sweden). Among these, Kerecis^®^ is particularly noted for its use in treating skin defects with ADM alone, without the need for subsequent skin grafts [7].

In addition, a novel reprocessed micronized ADM (mADM) was recently introduced. mADMs (CGderm Matrix^®^, CG Bio, Seoul, Republic of Korea) are a newer form of ADMs that have been freeze-dried and pulverized into thin, uniform sheets. This reprocessing reduces the metabolic burden of angiogenesis and allows rapid absorption and adherence to the wound surface. mADMs are enriched with collagen and extracellular matrix (ECM) components, which further enhance their wound-healing properties. Recently, the use of mADMs has increased owing to their versatility and effectiveness [8,9].

This study retrospectively analyzed the effect of mADMs on secondary intention healing in patients with complex soft-tissue defects in whom primary intention healing is difficult. The goal of this study was to assess the efficacy of mADMs in promoting secondary healing and improving clinical outcomes in these challenging cases.

## 2. Patients and Methods

### 2.1. Patients

This retrospective study reviewed the medical records of patients treated with sheet-type reprocessed mADMs (CGderm Matrix^®^, CG Bio, Seoul, Republic of Korea) between August 2022 and December 2022 at the Department of Plastic and Reconstructive Surgery of Soonchunhyang University Cheonan Hospital. This study adhered to the ethical guidelines of the Declaration of Helsinki and was approved by the Institutional Review Board (IRB) of Soonchunhyang University Cheonan Hospital (IRB FILE No. 2024-05-015).

### 2.2. Inclusion and Exclusion Criteria

Patients aged 18–90 years with full-thickness skin defects classified as complex wounds were included. Complex wounds are defined as wounds with significant tissue loss, infection, poor vascular supply, or in anatomically challenging locations where primary closure, skin grafts, or flap coverage are not feasible. Patients were treated exclusively with CGderm Matrix^®^ without other types of ADM. Patients were excluded if they were treated with additional types of ADMs besides mADMs; underwent surgical treatments such as primary closure, skin grafts, or flap coverage; were lost to follow-up during the treatment period; or had uncontrolled chronic conditions that could interfere with wound healing (e.g., severe uncontrolled diabetes, active malignancy) (Table 1). Patients with uncontrolled chronic conditions, such as severe uncontrolled diabetes, were excluded from the study. For the purpose of this study, uncontrolled diabetes was defined as having an HbA1c level above a certain threshold (e.g., 9.0%) without ongoing medical supervision or treatment for blood glucose control. However, patients with elevated HbA1c levels who were under active medical management and receiving treatment to stabilize blood glucose levels were included, as they were considered to be under adequate control despite elevated levels.

The following data were collected: age, sex, Ankle-Brachial Index(ABI), wound location, wound area, smoking history, history of reperfusion procedures (e.g., PTA), history of end-stage renal disease(ESRD), history of diabetes mellitus(DM), glycated hemoglobin (HbA1c), C-reactive protein(CRP), and Rutherford classification. The Rutherford classification system, established in 1986, is based on common risk factors and has been widely adopted for patient management and research (Table 2) [10].

### 2.3. Reprocessed mADM

**Figure 1:**
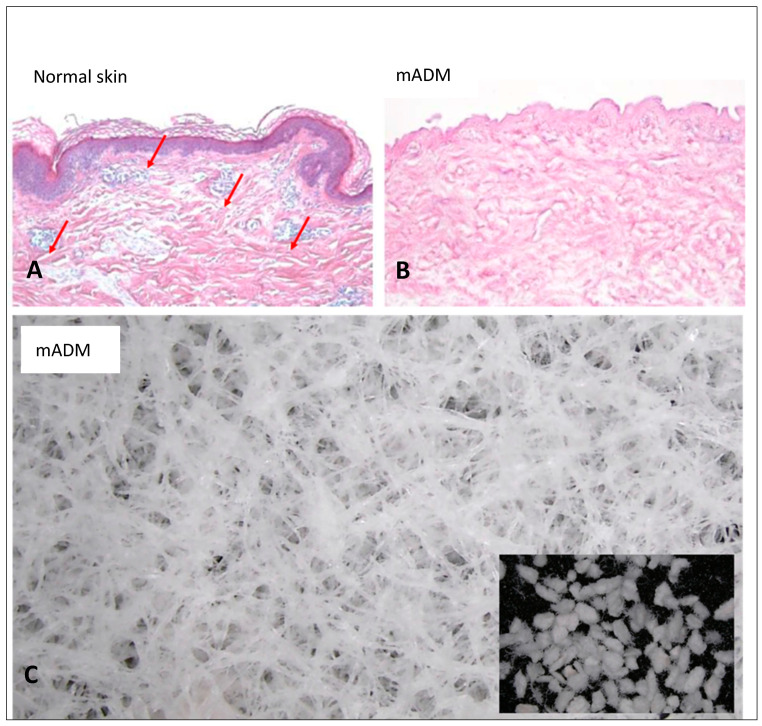
Histological and electron microscopy analysis of micronized acellular dermal matrix (mADM). (**A**) Histological comparison of the normal skin (left) using hematoxylin–eosin staining, where the purple color indicates the cell nuclei, and the pink color represents the extracellular matrix. (**B**) Histological image of the mADM showing the absence of the epithelial layer present in the normal skin and the lack of purple-stained cells within the matrix. (**C**) Scanning electron microscopy image of the mADM demonstrating the intricate porous structure, which facilitates cell migration and integration. The inset shows a higher magnification of the matrix’s uniform microstructure.

mADM (CGderm Matrix^®^) is an advanced form of ADM derived from human allogeneic dermis, that has been freeze-dried and pulverized into a thin, uniform sheet with a thickness of 0.7 mm. This process enhances its ability to support wound healing by enriching the wound with collagen and ECM components. Figure 1A illustrates normal skin architecture with intact epidermal and dermal layers. In contrast, Figure 1B displays the CGderm ADM, highlighting its scaffold structure, which is rich in collagen but devoid of cellular components. Figure 1C provides a scanning electron microscope image of the CGderm Matrix^®^, showcasing its finely woven ECM structure that acts as a scaffold to support cellular ingrowth and tissue regeneration. The inset in Figure 1C further details the microstructure of the pulverized ADM, highlighting its uniform particle size, which contributes to its ease of application and rapid absorption onto the wound surfaces. The reprocessing technique used for mADMs removes cellular and antigenic components, minimizing inflammatory responses and reducing the metabolic burden on the body’s natural angiogenic processes. By preserving the extracellular matrix (ECM), this reprocessed mADM provides a bioactive scaffold that supports vascular ingrowth with lower metabolic requirements. Furthermore, the presence of residual growth factors and cytokines within the ECM aids in the natural recruitment of wound-healing cells, enhancing angiogenesis with reduced metabolic demand.

### 2.4. Methods

**Figure 2:**
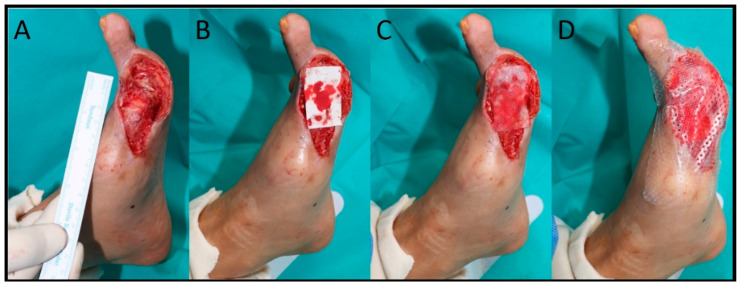
Application process of mADMs to a complex wound. The image was taken using a Canon EOS R6 Camera from a standard distance of 40 cm, with the camera positioned perpendicular to the wound. (**A**) Clinical photograph of a wound before the application of the mADM. A ruler is included in the image to provide a size reference. (**B**) Application of the mADM to the wound surface. (**C**) mADM hydrated and adhered to the wound, absorbing plasma. (**D**) Silicone barrier (Mepitel^®^ One) applied over the mADM to prevent detachment and absorption into NPWT devices. mADM, micronized acellular dermal matrix; NPWT, negative pressure wound therapy.

Before applying the mADM, the wound was documented using photographs, and after discharge, it was photographed at each outpatient visit. Clinical photographs of the patients were captured using a Canon EOS R6 Mark II Camera (Canon, Tokyo, Japan) from a standard distance of 40 cm (Figure 2A). The camera was positioned perpendicular to the wound to ensure consistent and accurate imaging. In addition, a ruler or standard-sized reference was included in each photograph to facilitate precise measurements. Using the recorded images, the wound area was measured using the ImageJ software (NIH, Bethesda, MD). For patients included in this study, sheet-type reprocessed mADMs were applied to the wound. When mADMs are applied to the raw surface of a wound, they rapidly hydrate and adhere, simultaneously absorbing plasma from the wound surface. mADMs exhibit a low metabolic burden concerning angiogenesis and can be rapidly resorbed and secured on the wound surface via plasma absorption (Figure 2B,C). The main concerns related to this approach were the potential for unforeseen detachment from the wound and absorption into negative pressure wound therapy (NPWT) devices. To mitigate these risks, a silicone barrier (Mepitel^®^ One; Mölnlycke Health Care, Gothenburg, Sweden) was used to cover the mADM to reduce the risk of loss due to shearing or friction (Figure 2D). Wound exudate was managed using a foam dressing or NPWT, as appropriate.

### 2.5. Statistical Analysis

The Shapiro–Wilk test was conducted to assess the normality of the variables. The Wilcoxon rank-sum test was used for nonparametric analysis of the median and interquartile range values. Furthermore, the significance of the changes in the wound area before and after applying the mADM was evaluated using the Wilcoxon signed-rank test. Spearman’s rank correlation was used to explore potential associations between variables and the rate of decrease in the wound area. A *p*-value < 0.05 was considered statistically significant. All analyses were performed using SPSS software (version 27.0; Chicago, IL, USA).

## 3. Results

### 3.1. Patient Enrollment and Exclusion

**Figure 3:**
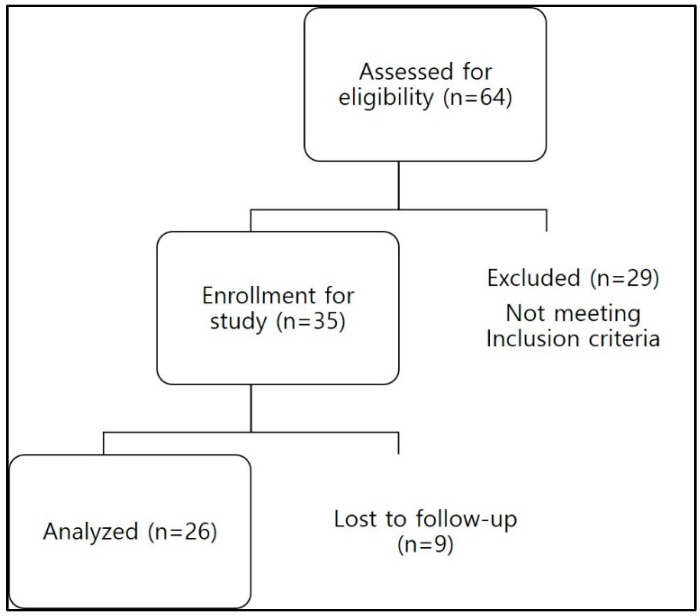
Flowchart showing the inclusion and exclusion of patients in the study. Initially, 64 patients were assessed for eligibility. Of these, 29 patients were excluded because they did not meet the inclusion criteria. The remaining 35 patients were enrolled in the study; however, 9 patients were lost to follow-up. Therefore, 26 patients were included in the final analysis.

Sixty-four patients treated with sheet-type reprocessed mADMs during the study period were enrolled. Twenty-nine patients were excluded, and nine patients were lost to follow-up; therefore, twenty-six patients were included in the final analysis (Figure 3).

### 3.2. Demographic and Clinical Characteristics

The demographic and clinical characteristics of the 26 patients included in the final analysis are summarized in Table 3. The majority of patients were male (92.3%), with a median age of 65 years, and 53.8% were aged ≥ 65 years. Most patients had a normal body mass index (BMI) (65.4% had a BMI < 25), and there was an equal distribution of smokers and non-smokers. A significant proportion of patients were diagnosed with diabetes (76.9%), with the majority (73.1%) having uncontrolled HbA1c levels. Most patients had a compromised vascular supply to their lower extremities, as indicated by the ABI of <1.2 in 78.9% of cases. Inflammatory response was observed in 42.3% of the patients with elevated CRP levels (≥5.00 mg/L). Additionally, 23.1% of the patients had ESRD and 11.5% underwent percutaneous transluminal angioplasty (PTA). The majority of wounds treated with mADMs were located on the foot (80.77%).

### 3.3. Classification Based on Complex Wound Type

The patients were further classified based on the type of complex wound, as presented in Table 4. Most patients had wounds with poor blood supply due to obstruction in the lower extremities (38.5%), where PTA had failed, followed by wounds with deep tissue defects exposing the bone, tendon, or articular surface (23.1%). Other categories included infected or inflamed wounds unsuitable for grafting (19.2%), including those with resistant bacteria, systemic vasculopathy (11.5%), and poor general condition preventing surgery (7.7%). This classification helps us to understand the distribution of different wound types and potential variations in response to mADM treatment.

### 3.4. Wound Area Reduction Results

The median (interquartile range) of the wound area measured before applying mADMs and at the last follow-up was 436.600 mm^2^ (1084.099) and 45.359 mm^2^ (368.446), respectively. The wound duration had a median of 45.5 days (10.2), and the follow-up period was 84.0 days (20.9). The percentage reduction in the area was 81.35% (54.699). The Wilcoxon signed-rank test indicated that the reduction in wound area was statistically significant (*p* < 0.01) (Table 5).

### 3.5. Correlation Analysis of Patient Variables and Wound Healing Outcomes

Upon analyzing the variables listed in Table 3, no statistically significant correlations were identified between the variables and the wound healing outcomes. Table 6 summarizes the Spearman’s rho and *p*-values for the correlations between age, BMI, ABI, CRP, HbA1c, and the rate of wound healing. None of these correlations were statistically significant.

### 3.6. Cases

#### 3.6.1. Case 1

**Figure 4:**
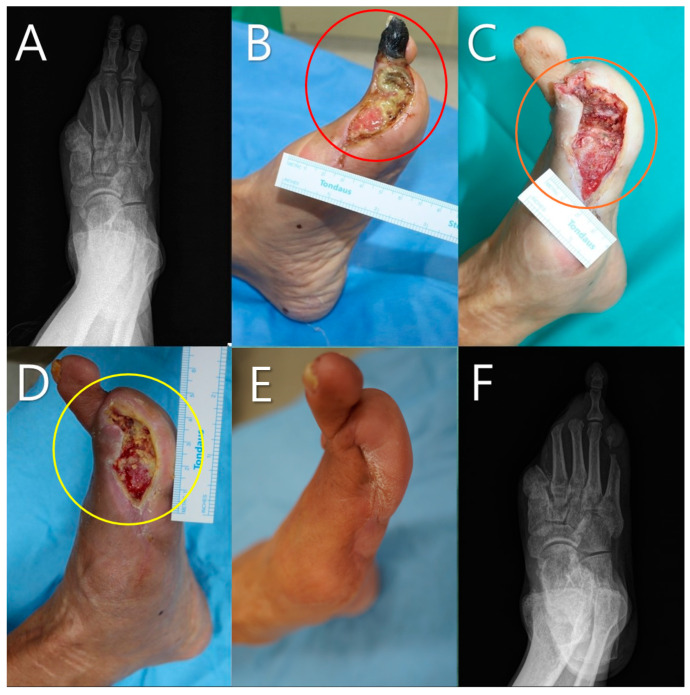
A 62-year-old male with a history of hypertension and diabetes. (**A**) Initial X-ray showing the extent of necrosis. (**B**) Pre-debridement image showing necrotic changes (red circle). (**C**) Post-debridement image showing the wound bed and poor tissue quality around the wound due to poor perfusion (orange circle). (**D**) Wound after 9 days following a total of four mADM applications, showing granulation tissue growth (yellow circle). (**E**) Wound after 12 weeks, showing successful secondary healing. (**F**) Final X-ray showing no signs of gas gangrene, pus accumulation, or osteomyelitis.

A 62-year-old male patient with a history of hypertension and diabetes presented with skin necrosis on the left foot (Figure 4). The patient had a complex wound due to total obstruction of the anterior and posterior tibial arteries, for which PTA was attempted but failed. Toe amputation and necrotic tissue debridement were performed because the necrotic changes (highlighted by the red circle in Figure 4B) necessitated minor amputation. The size of the defect measured before applying the mADM was 56 × 22 mm^2^. The surrounding tissue quality was poor due to inadequate perfusion, making primary revision challenging (highlighted by the orange circle in Figure 4C). The mADM was applied a total of four times to promote granulation tissue formation and facilitate wound healing. The patient was discharged 9 days after mADM application and received outpatient treatment. Granulation tissue began to grow into the wound (highlighted by the yellow circle in Figure 4D). Secondary healing was successfully achieved after 12 weeks without any complications.

#### 3.6.2. Case 2

**Figure 5:**
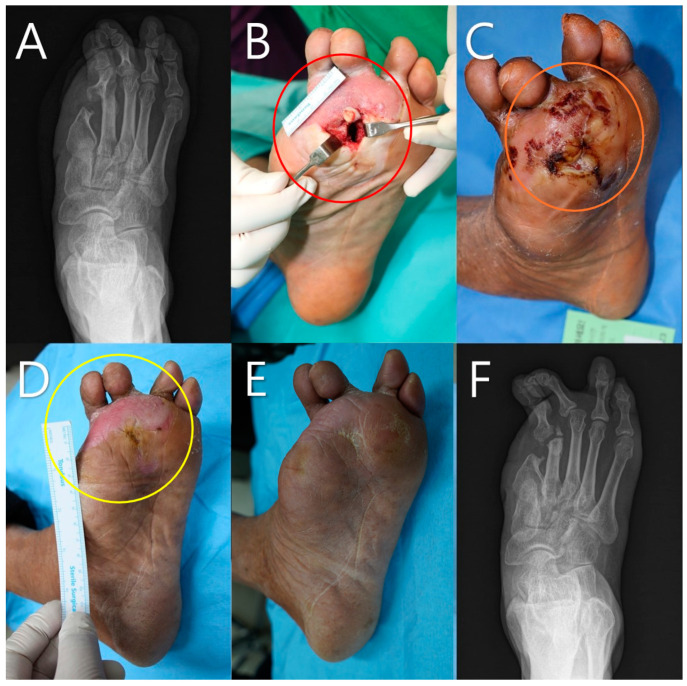
A 57-year-old male patient with a history of hypertension and diabetes. (**A**) Initial X-ray showing the extent of necrosis. (**B**) Intraoperative image showing escharectomy and necrotic tissue debridement, with red circle indicating the touching of bone and presence of *E. coli*. (**C**) Post-debridement image showing mild residual infection (orange circle) before micronized acellular dermal matrices (mADMs) application. (**D**) Wound after 20 days following a total of six mADM applications, showing significant healing with no signs of infection (yellow circle). (**E**) Wound at the 6-week outpatient follow-up, showing significant healing. (**F**) Final X-ray showing no signs of osteomyelitis.

A 57-year-old male patient with a history of hypertension and diabetes presented with a wet eschar on the plantar aspect of the left foot (Figure 5). The patient had a complex wound owing to the depth of the wound reaching the bone. The presence of *Escherichia coli* was identified from the wound cultures, and signs of osteomyelitis (highlighted by the red circle in Figure 5B). Escharectomy and necrotic tissue debridement were performed with NPWT. Despite debridement, mild signs of infection (highlighted by the orange circle in Figure 5C) were observed, making skin grafting or revision infeasible; therefore, the mADM was applied. The size of the defect measured before applying the mADM was 42 × 38 mm^2^. A total of six mADM applications were performed over the treatment period. The patient was discharged on the 20th day of serial mADM application. At the 6-week outpatient follow-up, it was confirmed that the wound had almost completely healed and appeared healthy with no further signs of infection (highlighted by the yellow circle in Figure 5D).

#### 3.6.3. Case 3

**Figure 6:**
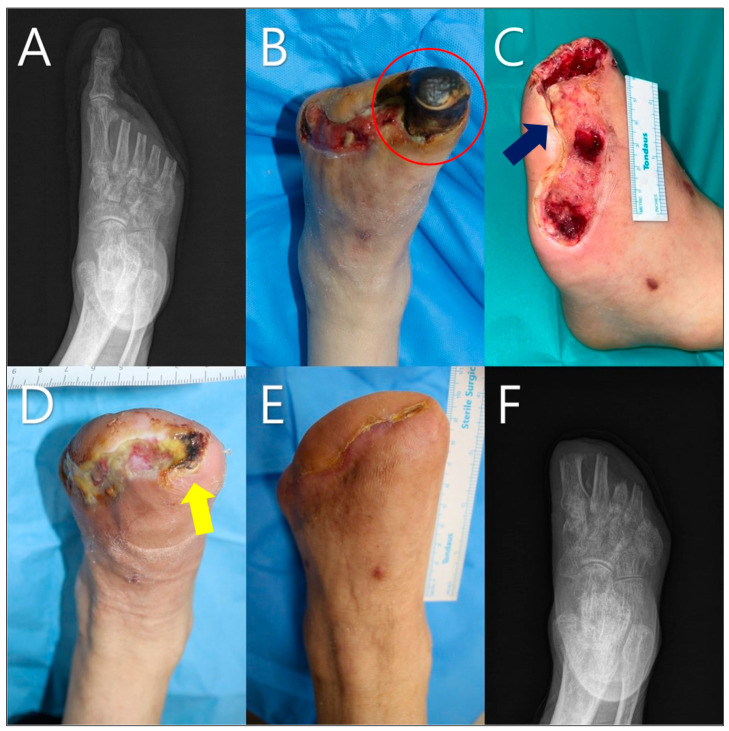
A 71-year-old male patient with a history of diabetes and angina. (**A**) Initial X-ray showing the extent of necrosis. (**B**) Pre-debridement image showing the necrotic great toe (red circle). (**C**) Post-debridement image showing exposed bone (blue arrow) before micronized acellular dermal matrices (mADMs) application. (**D**) Wound after initial mADM application, showing healing progress but with necrotic changes (yellow arrow). (**E**) Wound showing significant healing after a total of eight mADM applications, revision, and local flap. (**F**) Final X-ray showing no signs of bony and soft tissue complications, such as osteomyelitis, infection, or chronic inflammation.

A 71-year-old male patient with a history of diabetes and angina presented with diabetic gangrene in the left foot, accompanied by a foul odor and skin necrosis (Figure 6). The necrotic big toe (highlighted by the red circle in Figure 6B) required minor amputation. The patient had a complex wound with deep tissue defects exposing the bone and tendon. Open amputation and necrotic tissue debridement were performed; the blue arrow in Figure 6C indicates the exposed bone. The wound area measured before mADM application was 87 × 31 mm^2^. Despite mADM application, as seen in Figure 6D, the wound initially appeared to progress towards healing but eventually showed necrotic changes (highlighted by the yellow arrow). A total of eight mADM applications were performed as part of the serial application approach, along with revision and local flap procedures, to support complete healing. Healing was ultimately achieved, as shown in Figure 6E. Although this case was excluded due to exclusion criteria, it demonstrates the role of mADMs in preparing the wound bed for surgical approaches. Figure 6F shows no signs of bony or soft tissue complications such as osteomyelitis, infection, or chronic inflammation.

#### 3.6.4. Case 4

**Figure 7:**
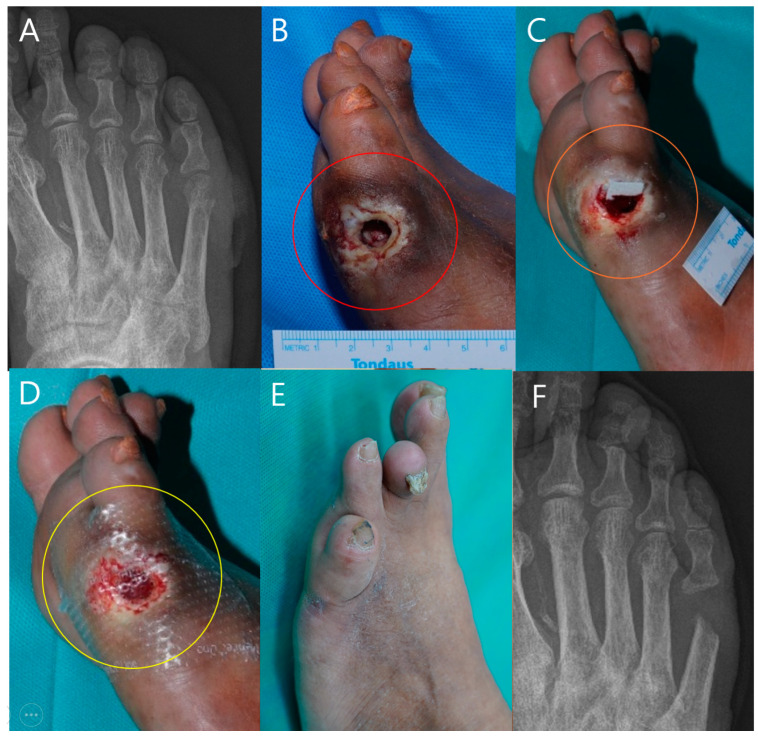
A 65-year-old male patient with a history of diabetes and peripheral arterial disease. (**A**) Initial X-ray showing the extent of bone involvement. (**B**) Clinical photograph prior to debridement, highlighting the necrotic tissue and bone exposure (red circle). (**C**) Post-debridement image showing the wound bed before micronized acellular dermal matrices (mADMs) application (orange circle). (**D**) Wound after 3 weeks following a total of five mADM applications, demonstrating significant granulation and healing progress (yellow circle). (**E**) Wound at the 8-week follow-up, showing re-epithelialization. (**F**) Final X-ray showing successful healing despite the presence of a bone defect, indicating effective re-epithelialization and wound closure.

A 65-year-old male patient with a history of diabetes and peripheral arterial disease presented with severe foot ulcer (Figure 7). The initial X-ray revealed extensive bone involvement (Figure 7A). Prior to debridement, clinical examination revealed necrotic tissue and exposed bone (red circle, Figure 7B). Following debridement, the wound bed was prepared for mADM application (orange circle, Figure 7C). A total of five mADM applications were performed over the 3-week treatment period as part of a serial application approach, resulting in significant granulation tissue formation and healing progress being observed (yellow circle, Figure 7D). At the 8-week follow-up, substantial re-epithelialization occurred (Figure 7E). The final X-ray confirmed successful wound closure despite the presence of a bone defect (Figure 7F).

#### 3.6.5. Case 5

**Figure 8:**
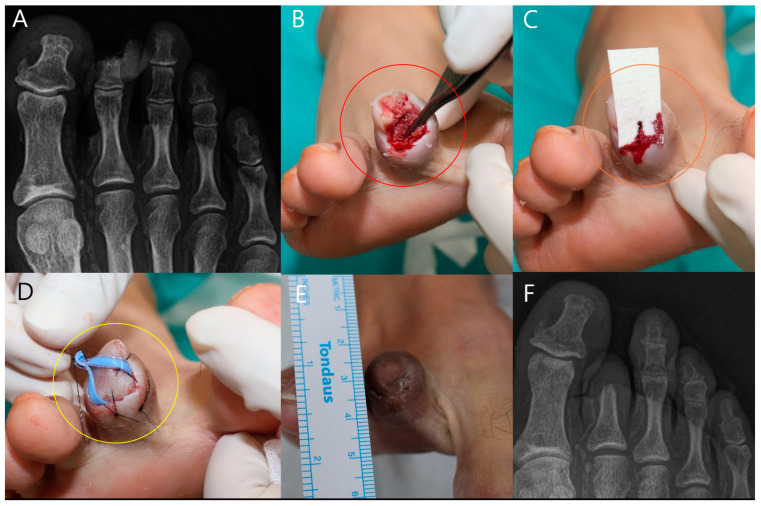
A 62-year-old female patient with a history of uncontrolled diabetes mellitus. (**A**) Initial X-ray showing bone resorption in the distal phalanx. (**B**) Intraoperative image after debridement, revealing significant bone exposure (red circle). (**C**) Single application of micronized acellular dermal matrices (mADMs) applied to the exposed bone (orange circle). (**D**) Wound closure achieved after mADM application, secured with sutures (yellow circle). (**E**) Six-week postoperative image showing substantial wound healing and epithelialization. (**F**) Final X-ray displaying successful bone regeneration and no evidence of osteomyelitis.

A 62-year-old female patient with poorly controlled DM presented with a toe ulcer characterized by bone resorption in the distal phalanx (Figure 8). Although her HbA1c levels indicated poorly controlled diabetes, the patient was under active medical supervision and receiving treatment to stabilize her blood glucose levels, meeting the study’s inclusion criteria despite elevated HbA1c. Following debridement, extensive bone exposure was evident (red circle, Figure 8B), and a single application of mADM was applied directly to the bone (Figure 8C). The postoperative management included securing the wound using sutures (yellow circle; Figure 8D). At the 6-week follow-up (Figure 8E), the wound showed significant healing, and the final X-ray (Figure 8F) confirmed bone regeneration with no signs of infection.

#### 3.6.6. Summary of Case

Table 7 provides a summary of key characteristics and outcomes for each representative case, including wound size, location, healing time, and additional relevant parameters.

## 4. Discussion

ADMs have been used as scaffolds in skin-grafting procedures for several decades to provide structural support and promote tissue regeneration [11]. Originally employed as a supplementary tool in skin grafts, ADMs have evolved as stand-alone dressing agents, demonstrating their versatility and efficacy in wound healing [12,13]. This shift in application underscores the growing recognition of ADMs’ potential to facilitate healing in complex wound scenarios without the need for additional surgical interventions [14,15].

The reprocessed mADM (CGderm Matrix^®^) is specifically designed to enhance wound healing by providing a robust ECM scaffold [4,16]. The process involves freeze-drying and pulverizing the ADM into a thin, uniform sheet, which reduces the metabolic burden of angiogenesis and allows for rapid absorption and adherence to the wound surface. mADMs are enriched with collagen, elastin, and other ECM components that play crucial roles in wound healing. Collagen provides structural support, whereas elastin enhances the tensile strength and elasticity [17,18]. Other ECM proteins promote cell migration, granulation tissue formation, and revascularization [19]. mADMs act as spacers, facilitating epithelization and angiogenesis, and effectively transform chronic and hard-to-heal wounds into acute wounds, thereby accelerating the overall wound healing process [20,21,22].

Collectively, these components contribute to the function of mADMs as wound accelerators. The inclusion of elastin, in particular, helps improve the tensile strength of the wound area, making the regenerated tissue more resilient [23,24]. Additionally, the rapid absorption and adherence properties of mADMs reduce the time required for wound coverage, thereby potentially decreasing the risk of infection and other complications [25]. By providing a supportive scaffold that facilitates the natural healing process of the body, mADMs enhance both the speed and quality of wound healing [26]. This makes them particularly suitable for complex wound cases in which traditional surgical interventions are not feasible or have failed. The versatility and effectiveness of mADMs highlight their potential as a valuable tool in modern wound-care management.

The present study demonstrated the efficacy of reprocessed mADMs in promoting secondary intention healing in patients with complex soft-tissue defects. The results indicate that mADMs can significantly reduce wound size and support the healing process, even in cases in which primary closure, skin grafts, or flap coverage are not feasible. This retrospective study included 26 patients with various types of complex wounds. Most of these patients presented with a compromised vascular supply, significant tissue loss, or infections that precluded the use of conventional surgical interventions. The application of mADMs in these patients resulted in a median wound area reduction of 81.35%, highlighting their potential for facilitating wound healing under challenging conditions.

Demographic and clinical characteristics of the 26 patients are summarized in Table 3. Most patients were male (92.3%), with a median age of 65 years, and had comorbidities such as diabetes (76.9%) and hypertension (53.8%). Many patients had poor a vascular supply (78.9% with ABI < 1.2) and elevated inflammatory markers (42.3% with CRP ≥ 5.00 mg/L). These characteristics highlight the complexity of the wounds treated in this study. Table 4 classifies patients based on complex wound types, revealing that a significant proportion (38.5%) had wounds with poor blood supply due to failed PTA. Other categories included deep tissue defects, infections, systemic vasculopathy, and poor general conditions that prevented surgery. This classification helps understand the distribution of different wound types and potential variations in response to mADM treatment.

The wound area measurements before and after mADM application are presented in Table 5. The median initial wound area was 436.600 mm^2^, and it was 45.359 mm^2^ at follow-up, with a median recovery rate of 81.35%. This significant reduction in the wound area illustrates the effectiveness of mADMs in promoting wound healing. Table 6 shows the correlations between patient variables and wound healing outcomes, with no statistically significant correlations. This highlights the complexity of wound healing and suggests that multiple factors influence the effectiveness of mADMs.

The case studies further illustrated the versatility and effectiveness of mADMs. A 62-year-old male patient with a history of hypertension and diabetes presented with skin necrosis on the left foot (Case 1, Figure 4). The patient underwent toe amputation and necrotic tissue debridement with a measured defect size of 56 × 22 mm^2^ before the mADM was applied. Despite the previous failure of PTA due to total obstruction of the anterior and posterior tibial arteries, the mADM was applied a total of four times as part of a serial application approach, tailored to support wound healing over the course of outpatient treatment. Serial application in this context refers to the repeated application of the mADM based on the clinical assessment of the wound’s healing progress and the condition of the surrounding tissue. The patient was discharged 9 days after mADM application and continued to receive outpatient treatment. Secondary healing was successfully achieved after 12 weeks without any complications. This case underscores the potential of mADMs in facilitating the healing of wounds with poor vascular supply, where primary surgical interventions are infeasible.

Another case involved a 71-year-old male patient with a history of diabetes and angina who presented with diabetic gangrene in the left foot accompanied by a foul odor and skin necrosis (Case 2, Figure 5). The patient underwent open amputation and necrotic tissue debridement, with a measured wound area of 87 × 31 mm^2^ before the mADM was applied. Despite the initial complications of wet debridement and necrotic changes, a total of six mADM applications, combined with revision and local flap procedures, ultimately led to successful healing. This case highlights the potential of mADMs in preparing wounds for further surgical intervention, even in complex scenarios in which immediate surgical closure is not feasible.

Another case (Case 3, Figure 6) involved a 71-year-old male patient with a history of diabetes and angina who presented with diabetic gangrene in the left foot accompanied by a foul odor and skin necrosis. The necrotic great toe (highlighted by the red circle in Figure 4B) required a minor amputation. The patient had a complex wound with deep tissue defects exposing the bone and tendon. Open amputation and necrotic tissue debridement were performed; the blue arrow in Figure 4C indicates the exposed bone. The wound area measured before mADM application was 87 × 31 mm^2^. Despite mADM application (Figure 4D), the wound initially appeared to progress towards healing but eventually showed necrotic changes (highlighted by the yellow arrow). However, healing was achieved after a total of eight mADM applications, revision, and local flap procedures (Figure 4). Although this case was excluded due to exclusion criteria, it demonstrates the role of mADMs in preparing the wound bed for surgical approaches. Figure 4F shows no signs of bony or soft tissue complications such as osteomyelitis, infection, or chronic inflammation.

In Cases 4 and 5 (Figure 7 and Figure 8), both of which involved male patients with diabetes and significant bone exposure due to foot ulcers, mADM application led to successful wound healing and re-epithelialization over several weeks. Despite the presence of bony defects, as observed in the final X-rays, the mADM effectively acted as a scaffold, facilitating granulation tissue formation and addressing dead spaces. These outcomes underscore the utility of mADMs in complex wound management, particularly in challenging environments involving bone exposure and soft tissue loss.

Even during surgery, the sheet-type reprocessed mADM has several distinct advantages. Initially, the thickness can be adjusted. The mADM used in this study had a thickness of approximately 0.7 mm. If a wound requires the use of a thicker ADM, its thickness can be easily adjusted by applying multiple layers of mADM. Its size can also be easily adjusted. Existing ADMs do not easily cover wounds wider than the size of the ADM. However, as mADMs are sheet-type ADMs, they can be easily shredded and deformed to fit the wound size. Finally, unlike ADMs, this type of reprocessed mADM does not require a fixation process. Because sufficient hydration is possible with only the blood in the wound bed without saline irrigation, it can be more easily attached to the wound. This can shorten the operation time.

The application of reprocessed mADMs in sheet format have demonstrated their efficacy in promoting secondary healing in challenging wound cases. When preceded by thorough restoration of blood circulation and meticulous wound bed preparation, the judicious utilization of mADMs has the potential to significantly expedite the overall wound healing process [16,27]. Notably, mADMs feature an ECM network architecture akin to established ADMs, characterized by a substantial presence of elastin and a remarkable tensile potency. These attributes collectively contribute to the augmentation of wound tensile strength, thereby enhancing wound healing outcomes [28,29].

Based on the results of this study, several indications for secondary intention healing may be recommended (Table 8). Secondary intention healing, which makes healing feasible without surgical intervention, is advisable for small wounds. It is also recommended when primary surgical interventions, such as flaps or grafts, are not feasible owing to infection or inflammation [30,31]. Additionally, it is suitable for patients who cannot undergo general anesthesia or prolonged surgery due to their overall condition. Delayed surgical intervention is another indication, particularly when a free or local flap is planned but delayed because of medical conditions or the need for further debridement to expose critical structures, making immediate skin grafting infeasible [32]. Secondary intention healing is also relevant when the vascularity is insufficient for surgical intervention because of severe obstruction or failed PTA, hindering procedures such as a free flap. It is applicable in cases of systemic vasculopathy such as Buerger’s disease, which makes flap procedures impossible [33,34]. Furthermore, it is suitable when sequential localized debridement and dressing are necessary due to poor wound perfusion or the patient’s medical condition. Lastly, it is recommended when previous attempts at free flaps, local flaps, or skin grafts have failed owing to various causes [35].

When comparing mADMs with other ADMs, it is clear that mADMs offer several distinct advantages [7,36,37] (Table 9). An mADM is easier to handle because of its pulverized form, which makes it suitable for application to contoured wounds and variable wound sizes. Unlike other ADMs, mADMs can be layered to adjust their thickness and rapidly hydrates, which simplifies their application. The versatility, ease of handling, and rapid hydration of mADMs make them particularly suitable for complex wound-healing scenarios. In addition, their composition, which includes substantial elastin and ECM components, supports wound tensile strength and overall healing [16].

This table provides a detailed comparison of various acellular dermal matrices (ADMs). It highlights the unique source, physical characteristics, clinical applications, and handling properties of these materials. This comparison underscores the distinct advantages of mADMs, such as their versatility in applications, ease of handling, and rapid hydration, which make them particularly suitable for complex wound-healing scenarios. The table also addresses potential disadvantages to offer a comprehensive understanding of the clinical utility of each product.

This study had several limitations. First, its retrospective design and reliance on medical records and 2-dimensional photographic data might have introduced biases and inconsistencies, particularly in the accurate measurement of wound areas and depths. Second, the absence of a control group limited our ability to draw definitive conclusions regarding the efficacy of mADMs compared with other treatments. Additionally, the small sample size of 26 patients restricted the generalizability of the findings. Finally, variations in patient compliance, underlying health conditions, and treatment protocols may have influenced the outcomes. Future prospective studies with larger sample sizes and control groups are required to validate these findings and optimize the use of mADMs in clinical practice.

Despite these limitations, this study provides valuable insights into the use of reprocessed mADMs for complex soft-tissue defects. These findings demonstrate that mADMs can significantly reduce the wound size and support the healing process, even in challenging cases in which conventional surgical interventions are not feasible. The versatility and ease of handling of mADMs make them a promising option for promoting secondary intention healing in complex wound management. Additionally, this study highlights the potential indications for secondary intention healing, offering a practical guide for clinicians in selecting appropriate treatment strategies for patients with complex wounds. These findings underscore the potential of mADMs in improving clinical outcomes and advancing the field of wound care.

Further research and controlled studies are required to expand these findings and optimize the use of mADMs in clinical practice. The retrospective design and lack of a control group in this study pose limitations, as does the use of 2-dimensional photographic data, which present challenges in accurately quantifying the wound area in complex anatomical regions. Future prospective studies with larger sample sizes and control groups are required to validate these findings and further explore the mechanisms of action of mADMs. Comparative studies between mADMs and traditional ADMs under similar wound conditions are also warranted to clarify their respective efficacy. Such research would be instrumental in developing optimal treatment strategies for complex wounds and further establishing the clinical utility of mADMs in secondary intention healing. Despite these challenges, this study provides a strong foundation for future research aimed at refining and enhancing the clinical application of mADMs in complex wound-healing scenarios.

## 5. Conclusions

mADMs demonstrated significant potential as an effective treatment for secondary intention healing of complex wounds, particularly when primary surgical interventions are not feasible. Their unique properties, including ease of handling, rapid hydration, and versatility make them a valuable tool for promoting wound healing. Further research is warranted to optimize their application and to fully understand their benefits in diverse clinical settings.

## Figures and Tables

**Table 1:** Inclusion and exclusion criteria.

Inclusion Criteria	Exclusion Criteria
Patients aged 18 to 90 years with full-thickness skin defects classified as complex wounds.	Patients treated with additional types of ADMs besides mADMs.
Patients treated exclusively with mADMs without other types of ADMs.	Patients who underwent surgical treatments such as primary closure, skin grafts, or flap coverage.
	Patients who were lost to follow-up during the treatment period.
	Patients with uncontrolled chronic conditions that could interfere with wound healing (e.g., severe uncontrolled diabetes, active malignancy).

Abbreviations: mADM, micronized acellular dermal matrix; ADM, acellular dermal matrix.

**Table 2:** Rutherford classification for chronic limb ischemia.

Grade	Category	Clinical Description	Objective Criteria
0	0	Asymptomatic—no hemodynamically significant occlusive disease	Normal treadmill or reactive hyperemia test
	1	Mild claudication	Completes treadmill exercise; AP after exercise > 50 mmHg but at least 20 mmHg lower than the resting value
I	2	Moderate claudication	Between categories 1 and 3
	3	Severe claudication	Cannot complete standard treadmill exercise, and AP after exercise < 50 mmHg
II	4	Ischemic rest pain	Resting AP < 40 mmHg, flat or barely pulsatile ankle or metatarsal PVR; TP < 30 mmHg
III	5	Minor tissue loss—nonhealing ulcer, focal gangrene with diffuse pedal ischemia	Resting AP < 60 mmHg, ankle or metatarsal PVR flat or barely pulsatile; TP < 40 mmHg
	6	Major tissue loss—extending above TM level, functional foot no longer salvageable	Same as category 5

Abbreviations: AP, ankle pressure; PVR, pulse volume recording; TM, transmetatarsal; TP, toe pressure.

**Table 3:** Demographic and clinical characteristics of the patients.

Characteristic	N (%)
Sex	
Male	24 (92.3%)
Female	2 (7.7%)
Age (years)	65.00 (18.00)
<65	12 (46.2%)
≥65	14 (53.8%)
BMI (kg/m^2^)	23.907 (5.004)
<25	17 (65.4%)
≥25	9 (34.6%)
Smoking	
Non-smoker	13 (50.0%)
Smoker	13 (50.0%)
DM	
None	6 (23.1%)
Diagnosed	20 (76.9%)
HbA1c	6.900 (2.3)
Controlled < 6.0	7 (26.9%)
Uncontrolled ≥ 6.0	19 (73.1%)
ABI	1.100 (0.24)
≥1.2	4 (21.1%)
<1.2	15 (78.9%)
CRP	3.590 (20.60)
<5.00	15 (57.7%)
≥5.00	11 (42.3%)
ESRD	
None	20 (76.9%)
Diagnosed	6 (23.1%)
PTA	
None	23 (88.5%)
Identified	3 (11.5%)
Location	
Sacral area	3 (11.54%)
Lower extremity (except foot)	2 (7.69%)
Foot	21 (80.77%)

Note: Data are presented as median (interquartile range). BMI, body mass index; DM, diabetes mellitus; HbA1c, glycosylated hemoglobin; ABI, ankle-brachial index; CRP, C-reactive protein; ESRD, end-stage renal disease; PTA, percutaneous transluminal angioplasty.

**Table 4:** Classification of the patients based on complex wound type.

Wound Type	N (%)	Characteristics
Poor blood supply due to obstruction in the lower extremities	10 (38.5%)	Wounds with poor vascular supply, often resulting in inadequate healing conditions. Includes cases where PTA has failed.
Deep tissue defects exposing the bone, tendon, or articular surface	6 (23.1%)	Wounds with significant tissue loss and exposure of deeper structures such as the bone or tendon.
Infected or inflamed wounds unsuitable for grafting	5 (19.2%)	Wounds complicated due to infection or inflammation, including resistant bacteria, making grafting difficult or impossible.
Systemic vasculopathy (e.g., Buerger’s disease)	3 (11.5%)	Wounds in patients with systemic vascular diseases affecting the overall blood supply and healing.
Poor general condition preventing surgery	2 (7.7%)	Wounds in patients with severe comorbidities or poor general health precluding surgical options.

PTA, percutaneous transluminal angioplasty.

**Table 5:** Wound area measurements.

	Median (IQR)
Initial wound area	436.600 mm^2^ (1084.099)
Wound duration	45.5 days (10.2)
Wound area at follow-up	45.359 mm^2^ (368.446)
Treatment period	35.00 days (16.00)
Follow-up period	84.00 days (30.9)
Recovery rate	81.359% (54.699)

IQR, interquartile range.

**Table 6:** Correlation between patient variables and wound healing outcomes.

	Spearman’s Rho	*p*-Value
Age	−0.163	0.427
BMI	−0.109	0.597
ABI	−0.074	0.765
CRP	−0.049	0.813
HbA1c	−0.079	0.702
Age	−0.163	0.427

BMI, body mass index; ABI, ankle-brachial index; CRP, C-reactive protein; HbA1c, glycosylated hemoglobin.

**Table 7:** Summary of case characteristics and outcomes.

Case	Age	Gender	Medical History	Wound Location	Wound Size (mm^2^)	mADM Applications	Healing Time (Weeks)	Follow-Up Period (Weeks)	Additional Procedures	Outcome
1	62	Male	Hypertension, Diabetes	Left Foot	56 × 22	4	12	16	None	Healing without complication
2	57	Male	Hypertension, Diabetes	Left Plantar	42 × 38	6	6	10	None	Healing without complication
3	71	Male	Diabetes, Angina	Left Foot	87 × 31	8	Excluded	12	Revision, Local Flap	Healing without complication
4	65	Male	Diabetes, Peripheral Arterial Disease	Left Foot	30 × 40	5	8	12	None	Healing without complication
5	62	Female	Poorly Controlled Diabetes	Right Second Toe	20 × 20	1	6	10	None	Healing without complication

**Table 8:** Indications for secondary intention healing.

Indication	Explanation
Small wound size	When the wound size is small, it is feasible to achieve healing without surgical intervention.
Infeasibility of primary surgical interventions due to infection or inflammation	When primary surgical interventions such as flap or graft are infeasible due to infection or inflammation.
Patient condition prohibits prolonged surgery or general anesthesia	When the patient cannot undergo general anesthesia or prolonged surgery due to their overall condition.
Delayed surgical intervention due to medical conditions or need for further debridement	When a free or local flap is planned but delayed due to medical conditions or the need for further debridement to expose critical structures, immediate skin grafting becomes infeasible.
Insufficient vascularity for surgical intervention	When vascularity is insufficient for surgical intervention due to severe obstruction or failed PTA, procedures such as free flap are hindered.
Systemic vasculopathy such as Buerger’s disease	When systemic vasculopathy (e.g., Buerger’s disease) makes flap procedures impossible.
Need for sequential localized debridement and dressing	When sequential localized debridement and dressing are necessary due to poor wound perfusion or the patient’s medical condition.

PTA, percutaneous transluminal angioplasty. This table outlines specific conditions under which secondary intention healing is recommended for complex wounds. Each indication is paired with a detailed explanation to clarify the clinical scenarios in which primary surgical interventions are not feasible, and secondary intention healing can be effectively utilized.

**Table 9:** Comparison of acellular dermal matrices.

	CGDerm^®^ (mADM)	Kerecis^®^	AlloDerm^®^	Integra^®^	MatriDerm^®^
Source Material	Human dermis (micronized, reprocessed)	Fish skin	Human skin	Bovine tendon	Bovine dermis and shark cartilage
Thickness	Adjustable (layering)	Variable	Fixed	Fixed	Fixed
Hydration	Rapid plasma absorption	Requires saline hydration	Requires saline hydration	Requires saline hydration	Requires saline hydration
Handling	Easy (thin, uniform sheet)	Moderate (requires rehydration)	Moderate (requires rehydration)	Moderate (requires rehydration)	Moderate (requires rehydration)
Metabolic Burden	Low	Low	Moderate	Moderate	Moderate
Angiogenesis Stimulation	High	Moderate	High	High	High
Structural Support	High	High	High	High	High
Clinical Applications	Secondary intention healing, wound bed preparation	Acute and chronic wounds, burns	Acute and chronic wounds, reconstructive	Acute and chronic wounds, burns	Acute and chronic wounds, burns, reconstructive
Advantages	Versatile, rapid absorption, easy handling, can be layered, quickly hydrates, fits contoured wounds easily	Omega-3 rich, anti-inflammatory	High integration, regenerative properties	Dual layer for epidermal and dermal regeneration	Contains glycosaminoglycans, promoting rapid vascularization and cell ingrowth
Disadvantages	Requires careful application to avoid shearing	Fish allergy concerns, requires rehydration	Requires rehydration, potential for immune response	Requires rehydration, potential for immune response	Requires rehydration, potential for immune response

mADM, micronized acellular dermal matrix.

## Data Availability

The dataset is available on request from the authors.
